# Inhibition of MMPs supports amoeboid angiogenesis hampering VEGF-targeted therapies via MLC and ERK 1/2 signaling

**DOI:** 10.1186/s12967-023-03954-6

**Published:** 2023-02-09

**Authors:** Anastasia Chillà, Cecilia Anceschi, Elena Frediani, Francesca Scavone, Tommaso Del Rosso, Giuseppe Pelagio, Antonio Tufaro, Giuseppe De Palma, Mario Del Rosso, Gabriella Fibbi, Paola Chiarugi, Anna Laurenzana, Francesca Margheri

**Affiliations:** 1 Department of Experimental and Clinical Biomedical Sciences “Mario Serio”, University of Florence, Viale G.B. Morgagni, 50, 50134 Florence, Italy; 2 Department of Physics, Pontifical Catholic University of Rio de Janeiro, Rio de Janeiro, RJ 22451-900 Brazil; 3 IRCCS Istituto Tumori Giovanni Paolo II Bari, Viale Orazio Flacco 65, 70124 Bari, Italy

**Keywords:** Angiogenesis, Protease inhibitors, Amoeboid migration, VEGF, Endothelial cells, ECFCs

## Abstract

**Background:**

In the past decades studies on anti-tumoral drugs inhibiting matrix metalloproteinase (MMPs) were disappointing. Recently, we demonstrated that mature endothelial cells (ECs) and endothelial colony forming cells (ECFCs) can switch between invasion modes to cope with challenging environments, performing the “amoeboid angiogenesis” in the absence of proteases activity.

**Methods:**

We first set out to investigate by ELISA if the inhibitors of the main protease family involved in angiogenesis were differently expressed during breast cancer progression. We used Marimastat, a broad-spectrum MMP inhibitor, as a means of inducing amoeboid characteristics and studied VEGF role in amoeboid angiogenesis. Thus, we performed invasion and capillary morphogenesis assay, morphological, cell signaling and in vivo mouse studies.

**Results:**

Our data showed that TIMP1, TIMP2, alpha2-antiplasmin, PAI-1 and cystatin increase in breast cancer serum of patients with primary cancer and lymph node positive compared to healthy women. In vitro results revealed that the most high-powered protease inhibitors able to induce amoeboid invasion of ECFCs were TIMP1, 2 and 3. Surprisingly, Marimastat promotes ECFC invasion and tubular formation in vitro and in vivo, inducing amoeboid characteristics. We observed that the combination of Marimastat plus VEGF doesn’t boost neither cell invasion nor vessel formation capacity. Moreover, inhibition of VEGF activity with Bevacizumab in the presence of Marimastat confirmed that amoeboid angiogenesis is independent from the stimulus of the main vascular growth factor, VEGF.

**Conclusions:**

We underline the importance to consider the amoeboid mechanism of endothelial and cancer cell invasion, probably responsible for the failure of synthetic metalloproteinase inhibitors as cancer therapy and tumor resistance to VEGF-targeted therapies, to set-up new drugs to be used in cancer therapy.

## Introduction

Judah Folkman was a pioneer in the study of angiogenesis and its related cancer therapy suggesting that “in order to stop tumor growth, one should attack its blood supply” [1]. Inhibition of angiogenesis has been, and still is a potential therapeutic paradigm for solid tumors [2–5].

The key mediator of tumor angiogenesis is the vascular endothelial growth factor, VEGF, secreted by cancer cells [6]. VEGF signals mainly through VEGF receptor 2 (VEGFR-2), which is expressed on vascular endothelial cells of neighboring blood vessels and on circulating bone marrow-derived endothelial progenitor cells [7, 8]. Gradients of soluble VEGF induce tip cells to sprout from a pre-existing vascular network, breaking down the surrounding extracellular matrix (ECM) and leading the growth of new vascular sprouts towards VEGF [4, 9]*.*

Most of the angiogenesis inhibitors currently used in the treatment of certain tumor types or eye diseases, rely on targeting VEGF itself or its receptors [10–12]. The first antiangiogenic drug was Bevacizumab, a humanized monoclonal antibody that binds to circulating VEGF-A isoforms preventing the interaction with their receptors. Since 2004, Bevacizumab was approved for the treatment of metastatic colorectal cancer, non-small-cell lung cancer, glioblastoma multiforme, ovarian cancer and renal cell carcinoma, generally in association with chemotherapy [13, 14]. Antiangiogenic therapy had a dramatic impact on the treatment of eye disease associated with aberrant growth of blood vessels [15, 16]. Conversely, in solid tumors, despite the initial expectations, the same therapies did not always yield the predicted results. During treatment with VEGF inhibitors, after an initial remission, tumors restart to grow and metastasize. This unstoppable progression is so far attributed to excessive increase of intratumoral hypoxia, blood flow alterations, angiogenic growth factors other than VEGF and activation of cancer stem cells [7, 17–19].

Proteolytic degradation of the ECM by proteases was always considered an absolute prerequisite for invasion and angiogenesis processes [20–22]. Over the decades, matrix metalloproteinases (MMPs) in particular, have been studied for their role in migration, angiogenesis and cancer progression [23]. MMPs are indeed a family of over 20 zinc-containing endopeptidases that, besides proteolytically cleaving ECM components, degrade the basement membrane and tumor surface, resulting in tumor cell infiltration into the near tissue. Recently it has become clear that MMPs’ role in the angiogenesis process is more complex than simply degrading ECM inducing endothelial cell invasion, thereby angiogenesis initiation. However, experimental evidence demonstrates that MMPs are also necessary for making available ECM-sequestered proangiogenic factors including cytokines and growth factors, exposing cryptic proangiogenic integrin binding sites in the ECM, helping to detach pericytes from vessels undergoing angiogenesis and cleaving endothelial cell–cell adhesions. However, MMPs can also contribute negatively to angiogenesis generating endogenous angiogenesis inhibitors by proteolytic cleavage of specific ECM components [24, 25].

In tumor angiogenesis, MMP-2 and MMP-9 have been shown to be critical during the “angiogenic switch”. Since the majority of produced VEGF is sequestered in the ECM deposited by tumor and stromal cells, the proteolytic release of angiogenic factors from tissue matrix, mainly mediated by MMP-2 and MMP-9, is essential for in vivo induced angiogenesis. Indeed, these MMPs are the main responsible for the release of VEGF. While MMPs can enhance the availability/bioactivity of angiogenic factors such as VEGF and its receptor VEGFR2, angiogenic factors can induce MMP expression in endothelial and stromal cells. In turn, the released VEGF, together with bFGF, directly regulate the microarchitecture and functions of the intratumoral vasculature, sustaining tumor cell intravasation and metastasis. Moreover, endothelial progenitor cells can be mobilized by VEGF and other cytokines from bone marrow into the circulation to reach the tumor site and contribute to tumor neovascularization [25, 26].

Individual cells can invade using mesenchymal or amoeboid invasive methods. Mesenchymal motility requires an elongated cell morphology, the formation of multiple focal adhesions, and the ability to degrade ECM by MMPs [27–31]. Recently, we first demonstrated that mature endothelial cells (ECs) and endothelial colony forming cells (ECFCs), migrate not only using the classical mesenchymal motility but can move and differentiate into vascular structures in vitro and in vivo also in the absence of protease activity [32]. Amoeboid motility does not usually utilize proteinases, and instead adapts cell shape to glide through preexisting gaps [33]. Thus, as well as cancer cells, endothelial cells can switch between invasion modes in order to cope with challenging environments [34, 35].

Human clinical studies with MMP inhibitors (MPIs) as antitumoral drugs were disappointing because patients experienced musculoskeletal pain, inflammation and tumor recurrence after an initial remission period [36–38]. In the previous study we induced amoeboid shift of endothelial cells using a physiological mix made by inhibitors of the main protease families involved in the angiogenesis: TIMP1, TIMP2, TIMP3, alpha2-antiplasmin, PAI-1 and cystatin. Here we evaluated the in vivo presence of the same protease inhibitors in breast cancer patients to show the existence of a specific mesenchymal-amoeboid transition step in tumor progression. Then, we tested in vitro groups of inhibitors to identify the main inducers of amoeboid transition in ECFCs. Marimastat, a broad-spectrum MMP inhibitor used in clinical [37–39], instead of inhibiting as previously thought, promoted the invasion and tubular formation inducing amoeboid characteristics. Thus, once the mesechymal-amoeboid shift was triggered by Marimastat, we used this model to explore VEGF role in this process inhibiting its activity with Bevacizumab.

Our data revise and expand the current concept on tumor angiogenesis uncovering a previously unknown protease-independent mechanism as responsible of the failure of MPIs as cancer therapy. Moreover, our findings explain tumor resistance to VEGF-targeted therapies as result of the onset of amoeboid cancer and endothelial cell behavior.

## Materials and methods

### Serum samples and patients’ characteristics

Serum samples from 67 patients with breast cancer were obtained from the Institutional Biobank (UNI EN ISO 9001:2015-certified) of the IRCCS Istituto Tumori “Giovanni Paolo II” of Bari, Italy, and stored at − 80 °C until used. The study was approved by the local Ethics Committee and patients gave their signed informed consent. Clinical parameters including age, stage, histological grade, and lymphatic metastasis were acquired from hospitalization records (Table 1).

**Table1:** Clinical characteristics of breast cancer patients

Characteristics	Lymph node status N− (%)	Lymph node status N+ (%)
Age (years)		
≥ 50	12 (26%)	4 (19%)
< 50	34 (74%)	17 (81%)
pT		
1	37 (80.4%)	10 (48%)
2	8 (17.4%)	11 (52%)
3	1 (2.2%)	0
Histological grade		
G1	9 (19.6%)	2 (9.5%)
G2	24 (52.2%)	10 (47.7%)
G3	13 (28.2%)	9 (42.8%)
pN		
0	46 (100%)	0
1	0	14 (66.7%)
2	0	2 (9.5%)
3	0	5 (23.8%)
Age (years) of healthy women		
> 50	8 (57.1%)	
< 50	6 (42.9%)	

### Protease inhibitor detection in patients’ serum

For quantitative measurement of human protease inhibitors in the patients’ serum, ELISA kits were used according to the manufacturer’s instructions. Standards and samples, were pipetted into the wells of the microplate on the bottom of which are bound specific monoclonal antibodies. After washing away any unbound substances, an enzyme-linked polyclonal antibody against each protease inhibitors and conjugated to horseradish peroxidase was added to the wells. A substrate solution was added and color change was measured spectrophotometrically at a wavelength of 450 nm ± 10 nm. Concentration of protease inhibitors was determined comparing the O.D. of samples to standard curve.

### Cell line, culture conditions and cell viability assay

ECFCs were isolated and grown as previously described [40]. Cell viability upon Marimastat (Merck) treatment was evaluated after 24 h by Trypan blue dye (Merck) exclusion assay. When Marimastat is tested, DMSO is always used as control. Human Retinal Endothelial Cells (HRECs) were gently provided by Dr. Lulli, Italy and grown in EGM-2 culture medium.

### 3D-invasion with Boyden chambers and scratch assay

Invasion was studied as previously described [32].

HRECs were seeded in 6-well plates at a density of 5 × 10^5^/well in complete medium and cultured to confluence. Cells were serum starved overnight in EGM-2. A yellow pipette tip was used to generate scratch wounds on the confluent cell monolayers. Cells were incubated at 37 °C for 48 h ± Marimastat. Microscopy was used to photograph cell migration to the scratch area and calculate the healing area of the wound by Image J.

### In vitro capillary morphogenesis

In vitro capillary morphogenesis was performed as described [32]. Where indicated, Rho activator II (5 µg/ml) and Rho inhibitor I (1 µg/ml) (Cytoskeleton) were added to cell suspension and maintained for the time of the experiment.

### Collagen degradation assay

The assay was performed as previously described [32].

### Cell proliferation assay

ECFCs were trypsinized, re-suspended, and seeded in 96-well plates coating with gelatin at a density of 1500 cells per well in 200 µl volume of EGM-2. Marimastat or VEGF were added respectively at a concentration of 10 µM and 25 ng/ml. After 3 days, cells were incubated with AlamarBlue® (Thermo Fisher Scientific) for 4 h. Fluorescence was measured at 530 nm excitation wavelength and 590 nm emission wavelength.

### In vivo Matrigel plug assay

All procedures involving animals were performed in accordance with the ethical standards and according to the Declaration of Helsinki and to national guidelines approved by the ethical committee of Animal Welfare Office of Italian Health Ministry and conformed to the legal mandates and Italian guidelines for the care and maintenance of laboratory animals.

Six 4-week-old male SCID beige mice (two for each experimental condition) were purchased from Charles River. VEGFA 50 ng/ml and Marimastat 50 µM and Heparin 50 U/ml were added to unpolymerized Matrigel at 4 °C at a final volume of 0.6 ml. Matrigel suspension was carefully injected subcutaneously into both flanks of mice using a cold syringe. Groups of four pellets were injected for each treatment. The six animals were subdivided as follows: two controls (Matrigel alone); two animals injected with Matrigel containing VEGF-A; two injected with Matrigel plus Marimastat. Five days after injection, plugs were removed, minced and diluted in water to measure hemoglobin content with a Drabkin reagent kit (Sigma). Vascularization was evaluated by sight taking a representative photograph of individual Matrigel plugs recovered at autopsy for the corresponding condition.

### Choroid sprouting assay

Choroid sprouting assay, usually used as an ex vivo model for studying microvascular angiogenesis, was performed testing Marimastat. Male C57BL/6J mice (age P20) were euthanized and eyes were immediately enucleated for dissection. After removing cornea and lens, the peripheral choroid-scleral complex was separated from the retina and cut into approximately 1 mm × 1 mm fragments. The choroid explants were immediately embedded in 30 μl growth factor reduced Matrigel in 24-well tissue culture plates. The explants were grown in DMEM supplemented with 10% FBS and 1% penicillin–streptomycin at 37 °C with 5% CO_2_. Six replicates were made for each experimental condition. The sprouting area was quantified using Image J under 4× magnification.

### RhoA and Rac1 activity assay

The assay was performed as previously described [32].

### siRNA ITGAV and CD44 knock-down

MISSION® esiRNA (Merck) were used according to the manufactures’s instructions, utilizing Lipofectamine 3000 transfection reagent (Thermofisher). Not-targeting siRNA pool constructs were used as negative control. Cells were incubated with transfection mix for 24 and 48 h.

### Immunofluorescence analysis

Cells were grown on coverslips in their culture conditions. Once at confluence, cells were treated with Marimastat 10 µM o/n. After treatment cells were fixed in paraformaldehyde according to routine immunocytochemistry methods. Immunofluorescence and confocal microscopy were performed as previously described [32].

### Western Blotting

Cell lysates, obtained after specific treatments (50 ng/ml VEGF; Bevacizumab 5 µg/ml; Marimastat 10 µM o/n), were resuspended in RIPA buffer (Merk) containing a cocktail of proteinase inhibitors (Merck) and the assay was performed as previously described [32]. Primary antibody used: RhoA and Rac1 (Mouse monoclonal 1:800, Millipore); uPAR (Mouse monoclonal 1:500 MON-R3, Invitrogen); p-KDR, KDR, pERK, ERK, pMLC2, MLC, WAVE and GAPDH (Rabbit polyclonal 1:1000, Cell Signaling).

### Statistical analysis

Unless otherwise stated, all the experiments were performed five times in duplicate for a reliable application of statistics. Statistical analysis was performed with GraphPad Prism5 software. Results are expressed as means ± SD. Multiple comparisons were performed by Anova and paired Student T test. Statistical significances were accepted at p < 0.05. (*p < 0.005, **p < 0.001, ***p < 0.0001).

## Results

### Quantification of protease inhibitors in breast cancer patients’ serum

**Fig. 1:**
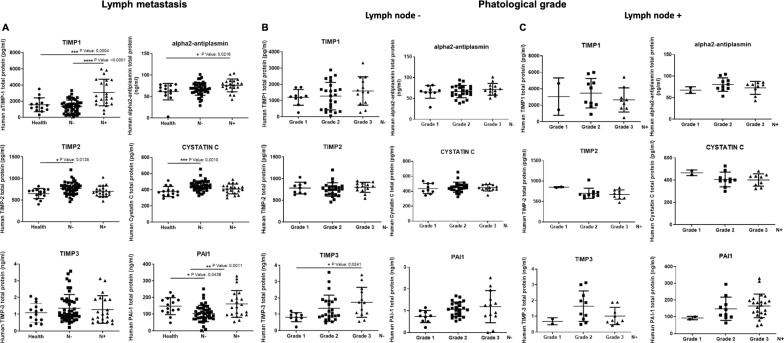
Protease inhibitor concentrations in the serum of breast cancer patients. Breast cancer patients were divided into 2 groups depending on invasive levels: lymph node-negative (n = 46) and lymph node-positive (n = 21). The lymph node-negative and positive patient serum was compared to samples from healthy donors (n = 14). TIMP1, TIMP2, TIMP3, PAI-1, alpha2-antiplasmin and cystatin in the patients’ serum were detected using commercial ELISA kits. **A** Comparisons between serum protease inhibitor concentrations in healthy people, lymph node-negative and lymph node-positive breast cancer patients. Each lymph node-negative (**B**) and lymph node-positive (**C**) group was then analyzed separately comparing the concentrations of each protease inhibitor for each different tumor grade (G1, G2 and G3). Each serum sample was tested in triplicate. Middle line in each group represents mean, lower- and upper-lines standard deviation (SD)

The amount of protease inhibitors was measured in the serum of breast cancer patients divided into 2 groups depending on invasive levels: lymph node-negative (n = 46) and lymph node-positive (n = 21). Each group was then analyzed separately according to the tumor grade (G1–G2–G3). Moreover, the lymph node-negative and positive patient serum was compared to samples from healthy donors (n = 14). For the quantitative measurement of human TIMP1, TIMP2, TIMP3, PAI-1, alpha2-antiplasmin and cystatin in the patients’ serum, the relevant human protease inhibitor ELISA kit was used. Figure 1 shows that all the inhibitors tested, except TIMP3, are significantly present in higher levels in cancer patients compared to healthy samples. In particular, as showed in Fig. 1A, TIMP1, TIMP2, cystatin and PAI-1 increase significantly in the lymph nodes− patients compared to healthy ones; only alpha2-antiplasmin in the lymph nodes+ compared to healthy ones; TIMP1 and PAI-1 increase also in the lymph node+ compared to the lymph node− group. Moreover, we analyzed each group, lymph node-negative and lymph node-positive, on the bases of the tumor grade G1, G2 and G3 (Fig. 1B for the lymph node− group and Fig. 1C for the lymph node+ group). In the lymph node-negative group, only TIMP3 resulted significantly increased in grade 3 compared to grade 1, instead we observed a not significant trend of increase of protease inhibitors in the higher grades compared to lowest. On the contrary, in the lymph node-positive group, both TIMP1 and TIMP3 showed an opposite trend with a not statistically significant reduction of their protein levels in the higher grades compared to lowest. Our results further strengthen what previously reported, confirming what was observed individually for TIMP1, TIMP2 and PAI-1, lately considered negative prognostic factors [41–43]. Moreover, here we show that even TIMP3, alpha2-antiplasmin and cystatin rather than decreasing, increase in cancer patient serum compared to healthy donors and some of them in metastatic patients compared to “in situ” tumor patients. These data suggest that the inhibition of the proteolytical activity, due to the increase of several protease inhibitors along tumor progression, could likely favor the amoeboid strategy inducing tumors to become more aggressive, thus justifying the failure of synthetic protease inhibitor therapy. To be noted that the discrepancy observed in the lymph node-positive group, for both TIMP1 and TIMP3, can be not real because more serum samples were needed to obtain statistically significant data. Moreover, we believe that tumor and endothelial cells can give a rapid response to the alterations of the microenvironment properties interchanging the amoeboid or mesenchymal type of movement according to the milieu found. This study indicates that measuring the amount of protease inhibitors in the serum can be essential to assess the susceptibility of single patients to a possible anti-amoeboid therapy.

### Use of Marimastat as amoeboid inducer on ECFCs

**Fig. 2:**
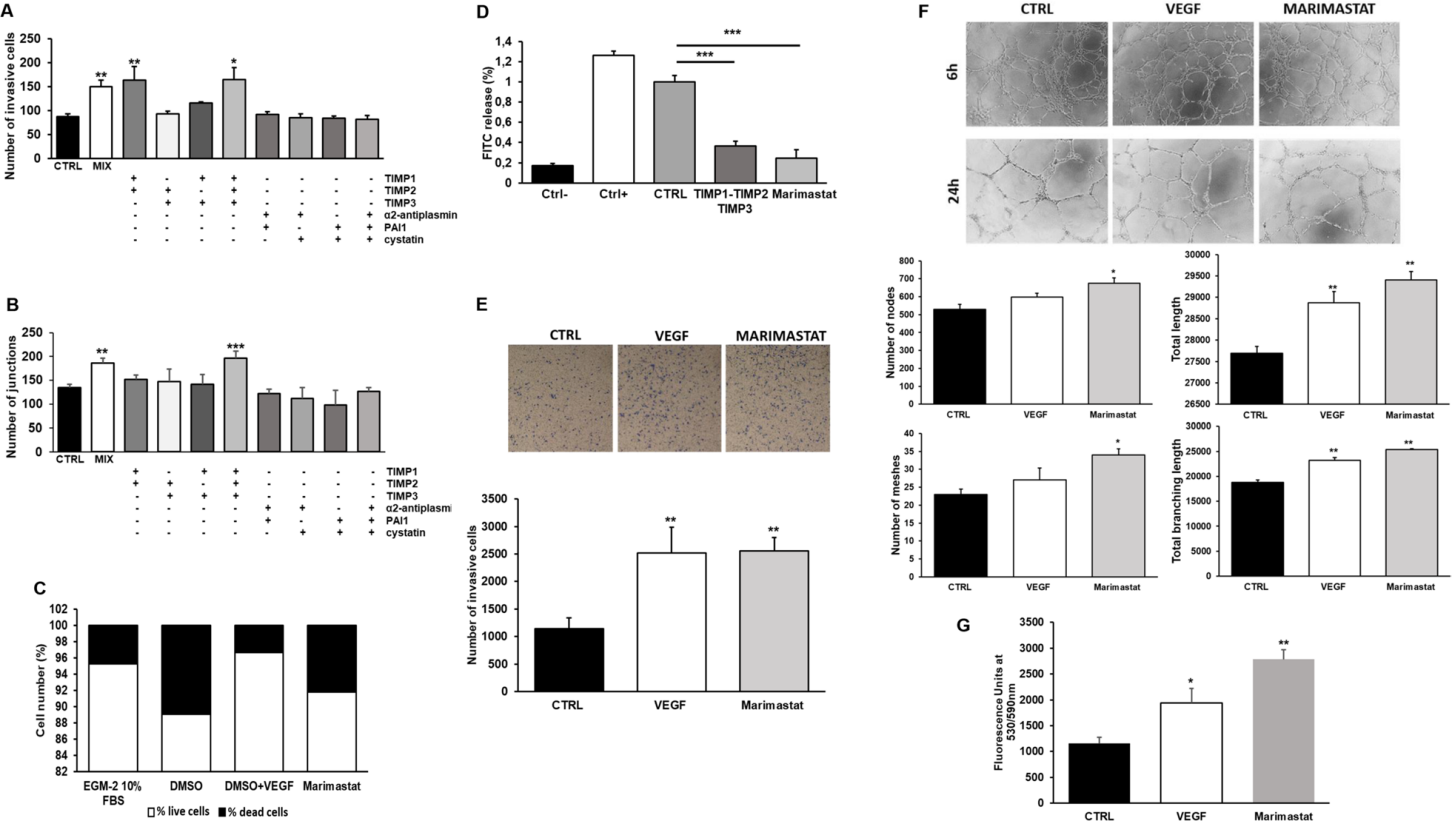
Evaluation of the effect of groups of protease inhibitors and Marimastat on in vitro invasion and angiogenesis. **A** Boyden chamber invasion assay through a thick Matrigel coating, in the presence of groups of the following protease inhibitors: TIMP1, TIMP2, TIMP3, alpha2-antiplasmin, PAI-1 and cystatin, and with all inhibitors mixed together (MIX) added to the Matrigel solution before polymerization. Histogram refers to quantification of Matrigel invasion assay obtained by counting the total number of migrated cells/filter. **B** In vitro angiogenesis measured by capillary morphogenesis at 24 h in the presence and in the absence of the same groups of inhibitors tested in **A**. Here it is shown the histogram representing the mean number of master junctions. Angiogenesis Analyzer Image J tool was used for the quantification of capillary network. Quantification was performed at 24 h after seeding and data are representative of measures obtained from at least nine photographic fields for each condition. **C** Cell viability was tested after Marimastat cell treatment for 6 (similar results not shown) and 24 h and evaluated by Trypan blue dye exclusion assay. The columns of histograms show in white the percentage of live cells and in black the percentage of dead cells. DMSO is to be considered the real control since it was used as Marimastat solvent. To be noted that in the next figures CTRL is to be considered as DMSO. **D** Histogram shows the collagenolytic activity of endothelial cells under mesenchymal (CTRL) and amoeboid conditions (TIMP1, TIMP2, TIMP3 or Marimastat), expressed as % collagen degradation with respect to the positive control obtained by addition of exogenous collagenase. Ctrl−: collagenolytic activity in the absence of cells and exogenous collagen; Ctrl+: collagenolytic activity in the absence of cells but in the presence of exogenous collagenase; CTRL: ECFCs; TIMP1-2-3 or Marimastat: collagenolytic activity of ECFCs in the presence protease inhibitors indicated. **E** Boyden chamber invasion assay through a thick Matrigel coating, in the presence of VEGF, here used as positive control, and Marimastat. The assay was performed as described in **A**. **F** Capillary network was quantified by Angiogenesis Analyzer Image J tool. Histograms represent the mean number of number of nodes, number of meshes, total length, and total branching length, respectively, at 24 h. Representative microphotographs (×10) of capillary-like structures at 6 and 24 h are shown. Data are representative of measures obtained from at least nine fields. **G** Effect of Marimastat on cell proliferation was quantified by AlamarBlue® assay and fluorescence was measured at 530 nm/590 nm. *n* = 3 independent samples

Figure 2A, B indicate that TIMP-1, TIMP-2 and TIMP-3 are the major contributors to amoeboid transition, promoting an intense invasion activity and tubular formation similarly to the full-range cocktail of all protease inhibitors mixed together. Based on these results we tested Marimastat, a chemical broad-spectrum MMP inhibitor used in clinics. After choosing the best concentration to use, according to literature and preliminary experiments, we first excluded its toxicity on ECFCs as shown in Fig. 2C. Then, collagenolytic activity of ECFCs was tested confirming a drastic inhibition of drug-induced collagen degradation, comparable to TIMP1, TIMP2 and TIMP3 effect (Fig. 2D).

Figure 2E–G show that Marimastat, instead of inhibiting, induced an increase of protease-independent cell invasion, vessel formation and cell proliferation like the stimulus of VEGF, here used as positive control. Histograms in Fig. 2F indicate that the number of nodes, meshes, total length and total branching length were significantly increased in Marimastat-treated ECFCs, and a more organized and mature capillary network was observed compared to untreated cells. Using this synthetic MMP-inhibitor, we were able to confirm that the switch of ECFCs from mesenchymal to amoeboid invasion strategy above described, was related to the inhibition of the ECM degradation by TIMPs, and not to any protease inhibition-independent activity of MMP inhibitors. To be noted that the residual collagenolytic activity shown by ECFCs treated with Marimastat does not justify the high number of invasive cells.

### The in vivo and ex vivo Marimastat effect on angiogenesis

**Fig. 3:**
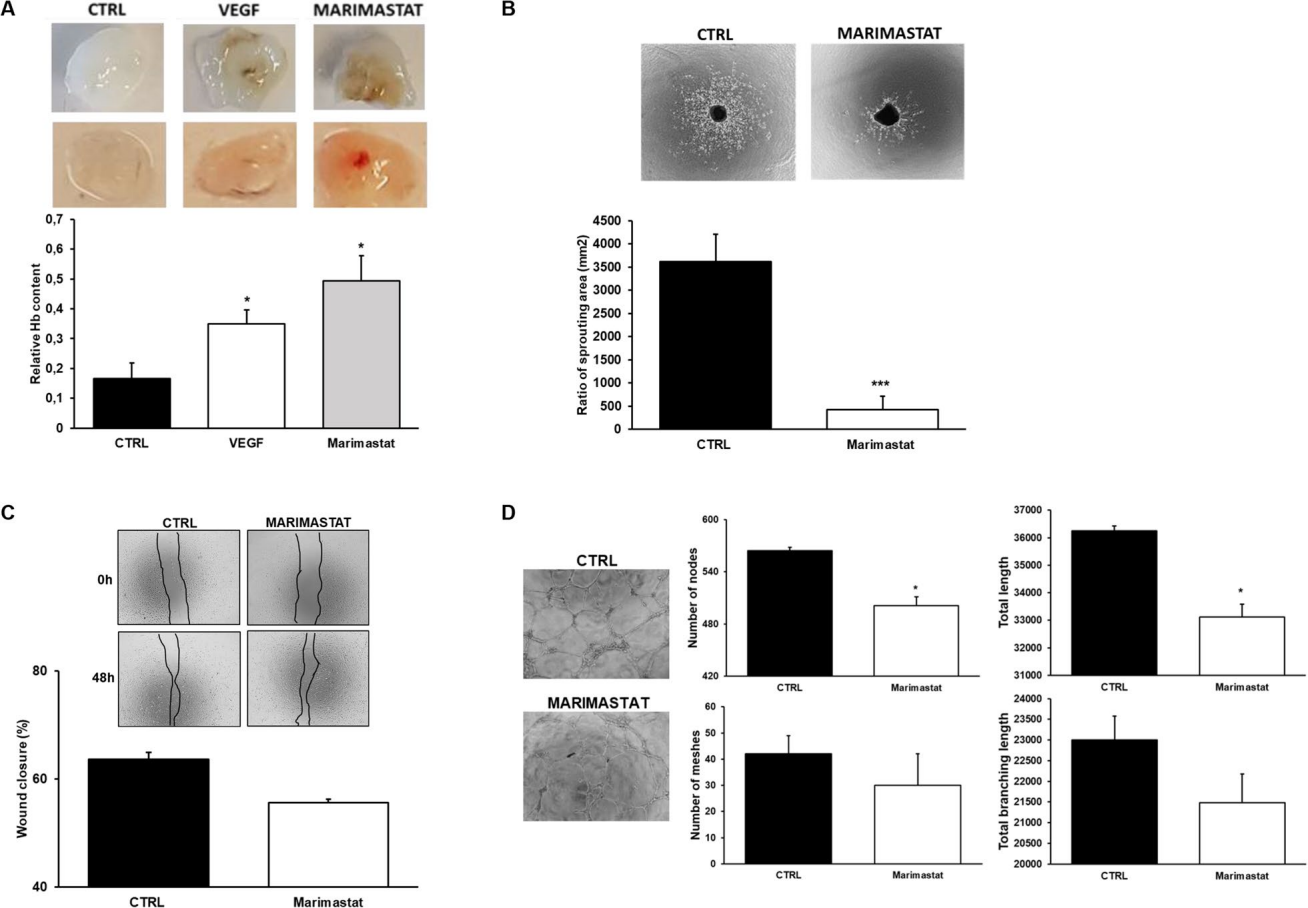
Effects of Marimastat on invasion and angiogenesis in vivo and ex vivo. **A** Angiogenesis in a Matrigel plug assay in SCID mice by the subcutaneosly addition of Matrigel containing heparin (50 U/ml) with and without VEGF and in the presence of Marimastat. In the first lane, representative photographs of individual Matrigel plugs recovered at autopsy for each condition shown. Angiogenesis was evaluated by hemoglobin (Hb) contents shown in the histogram below. **B** Mouse Choroidal Explant Assay: vessel outgrowth in a mouse choroid explant model in a 3D environment. Representative images of vessel growth after 6 days of incubation with Marimastat and quantification of the sprouting area by ImageJ. **C** Scratch assay was performed with HRECs in the presence of Marimastat. The histogram below indicates the quantification analysis of the scratch after 48 h measuring the wound closure. **D** Capillary morphogenesis of HRECs at 24 h in the presence and in the absence of Marimastat added to the Matrigel before polymerization. Representative microphotographs (×10) of capillary-like structures at 24 h are shown. Capillary network was quantified by Angiogenesis Analyzer Image J tool. Histograms represent the mean number of number of nodes, number of meshes, total length, and total branching length, respectively, at 24 h. Data are representative of measures obtained from at least nine fields. Results are the mean of 5 different experiments performed in duplicate and are shown as mean value ± SD. *p < 0.05; ***p < 0.0001 significantly different from control

To examine the effect of Marimastat on murine angiogenesis in vivo, matrigel plug assay was performed in SCID mice. Plugs recovered from mice showed that whereas control conditions displayed trace of vessels, Marimastat treatment stimulated angiogenesis (Fig. 3A).

Moreover, we used an ex vivo model for studying microvascular angiogenesis, thus performing a Choroid sprouting assay. Conversely, Marimastat inhibited vessel sprouting from mouse choroid in the 3D matrix (Fig. 3B). This discrepancy was probably due to the endothelial cell lineage used. For this reason, we tested the migration ability and capillary formation in vitro of HRECs. Figure 3C, D confirmed that Marimastat treatment inhibits, even slightly, the differentiation of HRECs in tubular structures and didn’t affect the migration ability without stimulating it, as showed testing ECFCs.

### Detection of the main molecules involved in the regulation of amoeboid angiogenesis

**Fig. 4:**
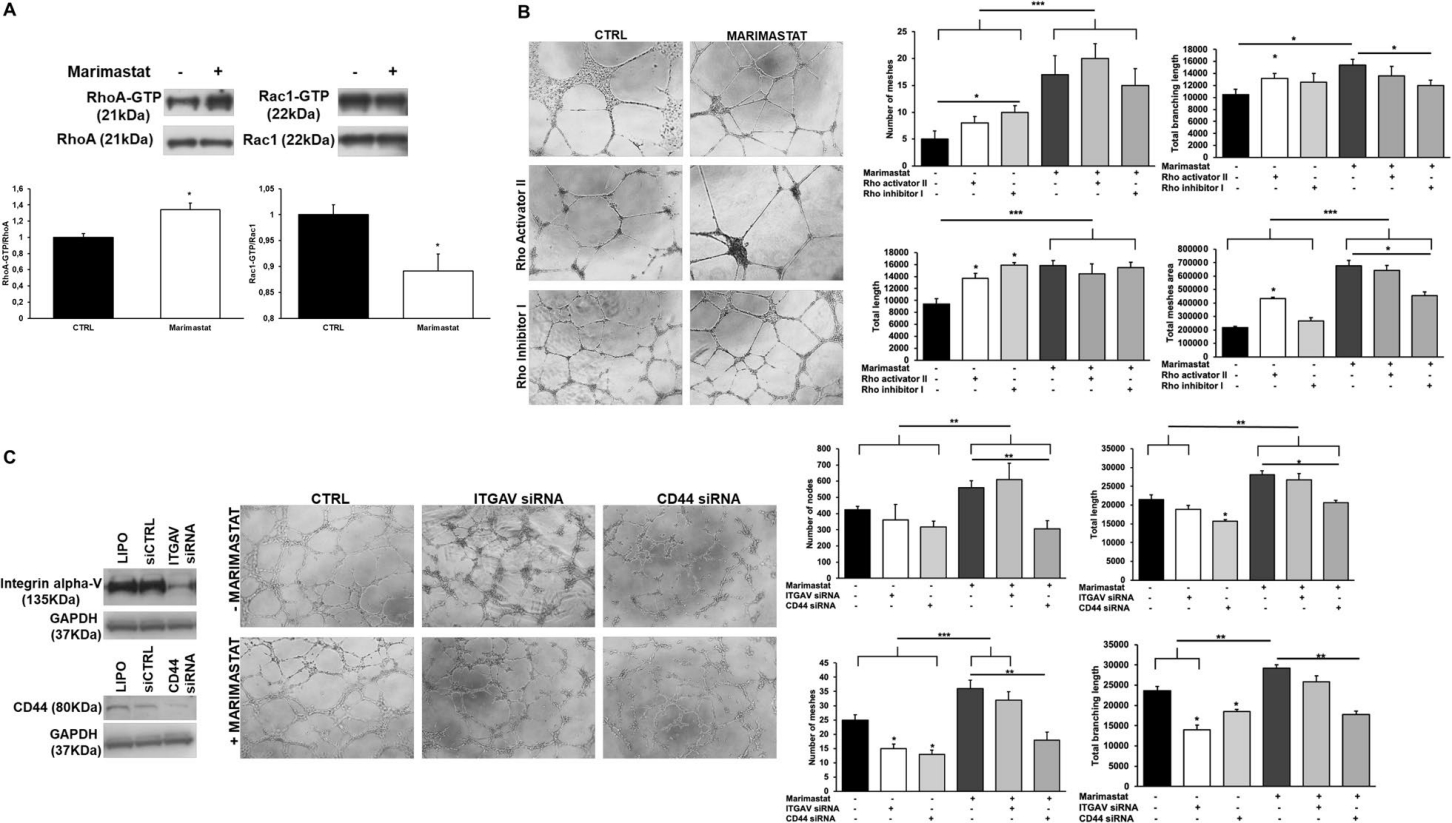
Involvement of RhoA, Rac1, αV integrin and CD44 on capillary morphogenesis after Marimastat treatment. **A** Western blotting of total and GTP-loaded forms of small Rho-GTPases RhoA and Rac1 in control conditions and after ECFC treatment with Marimastat. RhoA-GTP and Rac1-GTP, GTP-loaded forms of small Rho GTP-ases; RhoA and Rac, total un-loaded forms of small Rho GTP-ases, used as a reference loading control. Numbers on the left refer to molecular weights expressed in kDa. Histograms report band densitometry. Results are the mean of 5 different experiments performed in duplicate and are shown as mean value ± SD. *p < 0.05 significantly different from control. **B** In vitro angiogenesis of ECFCs in the presence of Rho Activator II (5 µg/ml) and Rho inhibitor I (1 µg/ml) was measured by capillary morphogenesis after 6 h in mesenchymal conditions (CTRL) and amoeboid conditions induced by Marimastat. Representative microphotographs (×10) of capillary-like structures are shown. Capillary network was quantified by Angiogenesis Analyzer Image J tool. Histograms represent the mean number of number of meshes, total length, and total branching length, and total meshes area respectively. Data are representative of measures obtained from at least nine fields. **C** The left panel shows western blotting analysis of integrin alphaV and CD44 after siITGAV and siCD44 treatment. LIPO: treatment of cells with the transfection reagent alone, lipofectamine. After silencing, ECFCs were subjected to capillary morphogenesis The assay was performed as usually and results are shown as described in **B**. Results are the mean of 5 different experiments performed in duplicate and are shown as mean value ± SD. *p < 0.05; **p < 0.001; ***p < 0.0001 significantly different from control or the experimental point indicated

Cell treatment with Marimastat induced an increase of activated RhoA, usually associated to amoeboid phenotype, and a decrease of activated Rac1 (Fig. 4A).

Figure 4B shows that RhoA activator in both mesenchymal and amoeboid conditions “accelerates” tube formation because the tubular structures are more mature compared to the control. Cells treated with Rho inhibitor in mesenchymal conditions, even if stimulated in tube formation compared to the control, as indicated by the histograms, resulted at an earlier step compared to cells treated with RhoA activator, with visible cell islands not yet differentiated. The inhibition of RhoA in the presence of Marimastat, instead, didn’t block completely tube formation but slow it down. Indeed, comparing the tube morphology at the same time points with that of Marimastat alone or Marimastat plus Rho activator, it results at an earlier step of maturation. Thereby RhoA plays a key role in the amoeboid cell motility but is not the only molecule driving it. Conversely, RhoA is not crucial in mesenchymal motility and even if inhibited, cells can still organize in tubular structures.

Sanz Moreno showed that MMP9 regulates amoeboid migration in a catalytic independent manner through regulation of actomyosin contractility via CD44 receptor in tumor cells [44]. In mesenchymal conditions, gene silencing of αVβ3 and CD44 showed respectively a regression in tube formation and an inhibition compared to their control, indicating the involvement of both molecules in the mesenchymal tube formation. The same siRNA tested in combination with Marimastat, instead, indicates that only CD44 is slightly involved in the amoeboid tube formation (Fig. 4C, right panel). The experiments were performed after validating the silencing activity of small interfering RNAs targeting integrin alphaV mRNA (siITGAV) and CD44 mRNA (siCD44), that produced an evident reduction of integrin alphaV and CD44 protein expression (Fig. 4C, left panel).

### VEGF appears irrelevant after ECFC treatment with Marimastat

**Fig. 5:**
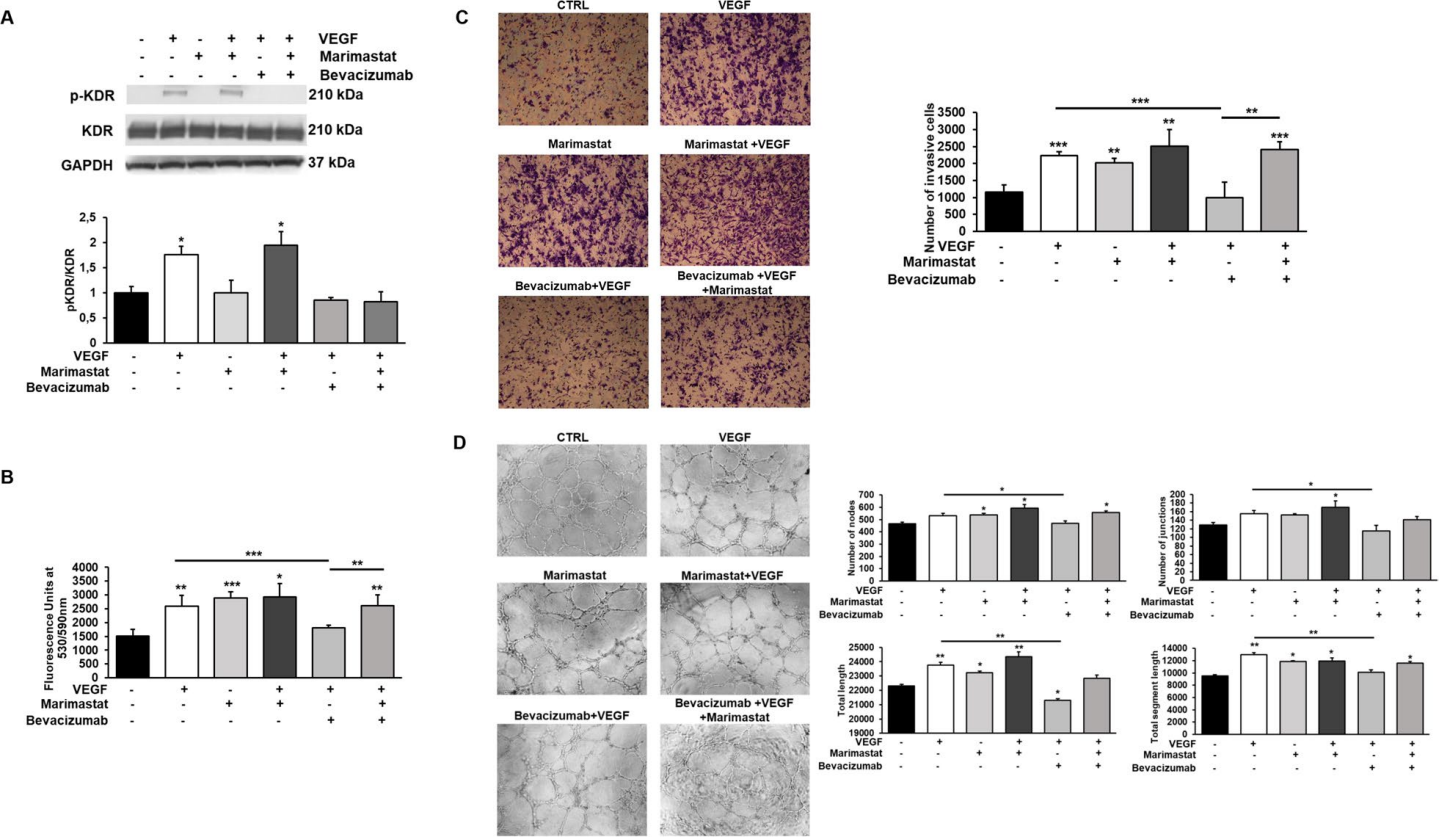
Effects of VEGF stimulation and its inhibition by Bevacizumab on ECFC proliferation, invasion and tubular structure formation. **A** Western blotting results show the phosphorylation of KDR in ECFCs after VEGF stimulation (50 ng/ml) and its inhibition by Bevacizumab (5 µg/ml) treatment in mesenchymal and amoeboid conditions. Numbers on the right refer to molecular weights expressed in kDa. Histograms report band densitometry. Results are the mean of 5 different experiments performed in duplicate, and are shown as mean value ± SD. **B** The effects of VEGF stimulation and its inhibition were also observed on cell proliferation (**B**), cell invasion (**C**) and in vitro angiogenesis (**D**). Cell proliferation was quantified by AlamarBlue® assay and fluorescence was measured at 530 nm/590 nm. *n* = 3 independent samples. Boyden chamber invasion assay and capillary morphogenesis were performed adding Marimastat to the Matrigel solution before polymerization and VEGF (25 ng/ml) ± Bevacizumab (5 µg/ml) in cell suspension. Representative microphotographs (×10) of migrated cell filters and capillary-like structures are shown. Histograms in **C** refer to quantification of Matrigel invasion assay obtained by counting the total number of migrated cells/filter. Capillary network was quantified by Angiogenesis Analyzer Image J tool. Histograms in **D** represent the mean number of nodes, number of junctions, total length, and total length of segments respectively. Data are representative of measures obtained from at least nine fields. Results are the mean of 5 different experiments performed in duplicate and are shown as mean value ± SD. *p < 0.05; **p < 0.001; ***p < 0.0001 significantly different from control or the experimental point indicated

The binding of VEGF to VEGFR-2 (or KDR) leads to receptor dimerization, kinase activation and autophosphorylation of specific tyrosine residues in the dimeric complex, inducing VEGF-mediated signal transduction. As shown in Fig. 5A, the analysis of Western Blotting experiments showed that cells treated with VEGF, both in the absence and in the presence of Marimastat, express the phosphorylated and active form of VEGF receptor-2. On the contrary, the presence of the VEGF inhibitor Bevacizumab, added concurrently with VEGF stimulation, inhibits KDR phosphorylation. These results therefore show that Bevacizumab inhibits the activity of VEGF, blocking the phosphorylation of its receptor, even in amoeboid conditions. Thus, we explored VEGF role in amoeboid angiogenesis inhibiting its activity using Bevacizumab.

Cell proliferation, invasion and tubular formation (Fig. 5B–D) resulted increased even in the presence of both VEGF and Marimastat, similarly to the effects of the growth factor or MMP inhibitor alone, without any additive effect. VEGF inhibition by Bevacizumab in the combined treatment didn’t change cell response, indicating that the effect shown is not due to the stimulus of VEGF but to the amoeboid shift mediated by Marimastat.

**Fig. 6:**
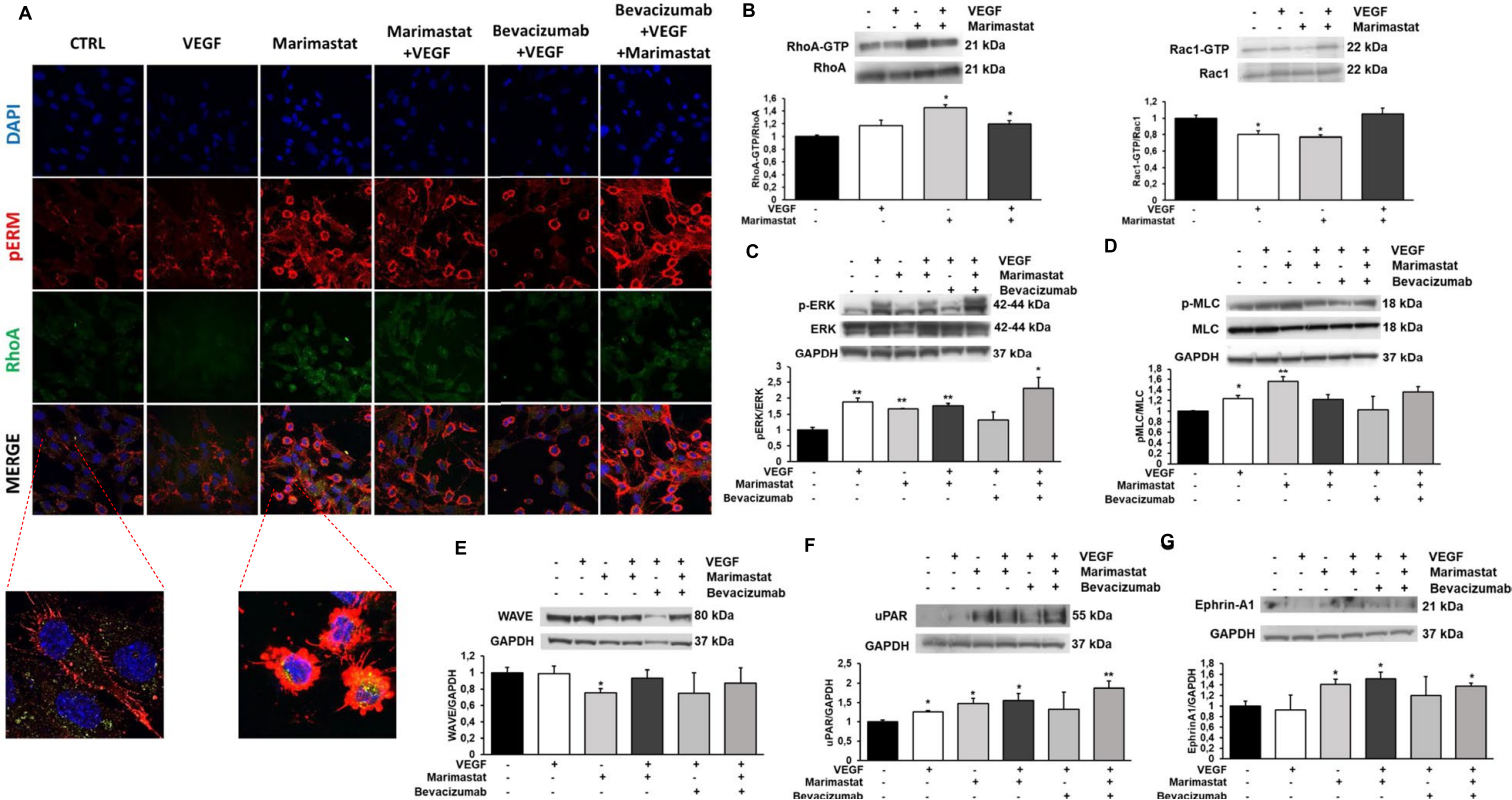
Effects of VEGF stimulation in Marimastat-induced amoeboid conditions on signaling molecules. **A** Confocal microscopy for pERM (red fluorescence) and RhoA (green fluorescence) under mesenchymal (CTRL) and amoeboid (Marimastat) conditions, in the absence and in the presence of VEGF (25 ng/ml) ± Bevacizumab (5 µg/ml). Morphological features of the mesenchymal (elongated) to amoeboid (roundish) transition of ECFCs are evident in the enlarged insets. Nuclear staining: DAPI (blue). Colocalization between pERM and RhoA: MERGE. Magnification ×40 for reference pictures and ×100 for enlarged insets. The shown pictures are representative of 30 different pictures for each experimental condition. **B**–**G** Western blotting results show the effects of VEGF, in mesenchymal and amoeboid conditions in the absence and in the presence of VEGF ± Bevacizumab, on the intracellular signaling molecules RhoA and Rac1, the phosphorylation of ERK and MLC2, WAVE2, uPAR and Ephrin-A1. Numbers on the right refer to molecular weights expressed in kDa. Histograms report band densitometry. Results are the mean of 5 different experiments performed in duplicate and are shown as mean value ± SD. *p < 0.05; **p < 0.001; ***p < 0.0001 significantly different from control

As previously observed, the type of movement adopted by cells is also closely related to their morphology. Figure 6A shows the confocal immune-fluorescence analysis of RhoA and p-ERM in ECFCs. RhoA, as described above, is a small GTPase that plays a fundamental role in the amoeboid movement, but it also takes an active part in the mesenchymal movement, while ERM is a family of proteins belonging to the cytoskeleton, in particular consisting of Ezrin, Radixin, and Moesin that are involved in the actin filament/plasma membrane interaction as cross-linkers. Activation of RhoA is able to induce phosphorylation of ERM proteins. RhoA and p-ERM proteins can be considered, together with F actin, as structural constituents of blebs. The images captured by the confocal microscope show that pERM appears mainly situated at apical structures in mesenchymal ECFCs, in particular on the membrane protrusions of the cell edge such as filopodia. In amoeboid ECFCs, the phosphorylated form of ERM appears overexpressed with aberrant localization in bleb structures. Figure 6A also shows that RhoA expression is increased in amoeboid conditions. Moreover, since ERM proteins interact with actin filaments, cell visualization at confocal microscopy revealed the rounded and blebbing morphology assumed by cells treated with Marimastat compared to the elongated morphology of cells under mesenchymal conditions.

In addition to the small GTPases RhoA and Rac1 (Fig. 6B), intracellular pathways related to angiogenesis were analyzed, in particular with the aim to test whether VEGF stimulation in Marimastat-induced amoeboid conditions triggers the activation of any signaling molecules. We observed an increase in ERK1/2 phosphorylation after Marimastat treatment especially in the presence of VEGF, showing that ERK pathway plays a relevant role in amoeboid conditions (Fig. 6C). AKT pathway instead does not seem to be involved in EC amoeboid movement since its phosphorylation levels do not increase (data not shown). We investigated the phosphorylation of Myosin Light Chain 2 (MLC2), that is associated with amoeboid movement, and we found elevated levels in Marimastat treated cells (Fig. 6D). Conversely, WAVE, that is responsible for downregulation of amoeboid motility and therefore of actomyosin contractility and membrane blebbing, is decreased in the presence of Marimastat (Fig. 6E). A similar balance between MLC2 and WAVE was found in the previous study where a physiologic protease inhibitor MIX was used to induce the amoeboid shift. The urokinase-mediated plasminogen activation system is a complex system of serine proteases strongly involved in angiogenesis. uPAR expression, in fact, is increased in EC with Marimastat as well as Ephrin A1 expression, that it is known to promote RhoA activation and amoeboid transition in tumor cells (Fig. 6F, G). The combination of Marimastat and VEGF does not further trigger the activation of the analyzed signaling molecules compared to their expression in cell treated with Marimastat alone. Moreover, VEGF inhibition with Bevacizumab in amoeboid conditions, does not modulate any molecule expression that appears comparable to that one mediated by the combination of Marimastat and VEGF mixed together.

## Discussion

Cancer progression is not only dependent on genetic or epigenetic alterations in cancer cells but is closely related to tumor microenvironment (TME) [45]. TME is subjected to continuous changes in composition in response to environmental pressures and anticancer therapies. For instance, changes in the mechanical properties of the tumor surrounding ECM and the development of tumor-specific vasculature generate a permissive microenvironment to cancer progression [46, 47].

Originally, proteases were believed key mediators of invasion and angiogenesis processes considering the proteolytic degradation of the ECM an absolute prerequisite [20, 21]. Therefore, physiological protease inhibitors, such as TIMPs, were deemed the main regulators of the ECM remodeling by inhibiting proteolytic activity in both normal and disease states. This evidence led to the development of synthetic MPIs, such as Marimastat, for cancer therapy. The initial enthusiasm for clinical application of MPIs was unexpectedly curtailed by their failure in early clinical trials, mainly due to the low oral bioavailability, lack of efficacy and toxicity [36]. More recently, TIMPs have been shown to possess different and unexpected MMP-independent functions [48]. In fact, some of protease inhibitors are validated or promising prognostic candidates in cancer for their tumor-stimulatory functions. For instance, it has been reported an association in breast cancer between high serum levels of TIMP1 and TIMP2 and several parameters indicative of tumoral aggressiveness and lower overall survival [41, 43]. It can seem paradoxical because elevated levels of TIMPs should result in decreased MMP proteolytic activity and consequently would prevent tumor progression and thus be related with good outcome in patients with cancer. Among the MMP-independent functions of TIMPs, recent studies have described their interaction with cell surface receptors, thereby mediating cell growth, proliferation, and apoptosis [49]. Even PAI-1 is considered a prognostic biomarker in breast cancer, as testified by high levels found in tumor extracts. A possible mechanism, so far accepted, responsible of this alternative behavior is the enhancing of angiogenesis by protecting ECM from its excessive degradation thus preserving a scaffold for endothelial cells migration and capillary formation. In addition, PAI-1 is also able to block apoptosis, promoting consequently cancer progression enhancing cell survival [42].

Our observations suggest that, besides the explanations commonly offered, a new mechanism of endothelial cell invasion and vessel formation plays a crucial role in the responsiveness of tumors to MPIs, and it is probably responsible for their failure. In our previous report we described for the first time the protease-independent “amoeboid angiogenesis” [32]. In this study we first set out to investigate if the inhibitors of the main protease family involved in the angiogenesis process were differently expressed during breast cancer progression. We used breast cancer because MMP inhibitor treatments provided no benefit in both early and late stages and even the positive responses to VEGF inhibition found in metastatic colorectal or lung cancer were not matched in metastatic breast cancer [50, 51]. As described above, it has been reported that high serum levels of TIMP1, TIMP2 and PAI-1 are correlated with lower overall survival and are then considered negative prognostic factors [41–43]. However, most reports have focused on the role of the singular inhibitors on breast cancer progression, and we first suggest that various inhibitors increase and are involved in tumor progression favoring the amoeboid strategy, thus justifying the failure of MPIs. In particular, our data showed that all inhibitors above tested, except TIMP3, are significantly present in higher levels in cancer patients compared to healthy samples. In particular, as showed in Fig. 1A, TIMP1, TIMP2, cystatin and PAI-1 increase significantly in the lymph nodes− patients compared to healthy ones; only alpha2-antiplasmin in the lymph nodes+ compared to healthy ones; TIMP1 and PAI-1 increase also in the lymph node+ compared to the lymph node− group.

Then, we tested in vitro groups of the protease inhibitors described to identify the main inducers of amoeboid transition in ECFCs. The tissue inhibitors of metalloproteinase had the highest impact among the mix components. Based on these results, we decided to test the effect of Marimastat, a broad-spectrum MMP inhibitor used in clinical, on ECFCs. Marimastat acts by mimicking the substrate of the MMPs enabling their downregulation, especially of MMP-1, MMP-2, MMP-3, MMP-7, MMP-9 and MMP-14 [39]. Promising in vitro and in vivo animal results from different studies led to intensive efforts to use Marimastat in clinical to inhibit cancer progression. Surprisingly, the disappointing results shown in clinical trials [36] led no one to investigate Marimastat effect on TME, in particular its impact on endothelial cells. Some reports showed an inhibition of tumor angiogenesis but just at an early stage. In different studies Marimastat has been reported to inhibit 3D cancer cell migration in monoculture [52] but just one study showed the failure in inhibiting cancer cell migration of MDA-231 if co-cultured with fibroblasts [53]. This was the first one suggesting the importance to incorporate TME components in in vitro models providing a possible alternative explanation of the discrepancy between the successful MMP inhibitors in vitro results and the failure in clinical trials. In a paper published in 2002 on Cancer Research, reporting the anti-angiogenic role of an aromatic sulfonamide derivative, it has been shown that HUVEC treatment with Marimastat didn’t inhibit neither proliferation nor capillary tube formation [54]. A year later another study showed similar results also testing Marimastat on sprout formation from aortic explants, reporting no inhibition [55]. Despite these evidences nobody pointed out the importance to investigate the response of endothelial cells to MMP inhibition especially on the basis of the failure of Marimastat and the other MPIs in clinical. Here, morphological studies, invasion assay, capillary morphogenesis assay, proliferation assay, biochemical and in vivo evidence confirmed that Marimastat, instead of inhibiting as previously thought, promotes the invasion and tubular formation of ECFCs, inducing a mesenchymal to amoeboid shift as evidenced by RhoA-GTP activation. The in vivo matrigel plug assay showed that Marimastat was able to induce murine vessel formation, instead the ex vivo choroid sprouting assay revealed opposite results because Marimastat inhibited vessel sprouting from mouse choroid in 3D matrix. This discrepancy was probably due to the endothelial cell lineage used as confirmed from the inhibition, though slight, of in vitro angiogenesis after retinal endothelial cells treatment with Marimastat.

In addition to the indirect approach involving MPIs, other strategies were used to block tumor progression by inhibition of tumor angiogenesis. Especially to be highlighted is a more direct approach against the most potent pro-angiogenic factor, VEGF. For different reasons, even VEGF inhibitors used in clinical trials have not met the exciting expectations [11–13]. Here, we used Marimastat to induce mesenchymal-amoeboid transition as model to study VEGF role during this process. Our results reveal that during Marimastat treatment and after VEGF inhibition by Bevacizumab, ECFCs do not seem to undergo any changes in amoeboid features. These data lead us to rule out that the functional stimulation as well as the activation of signaling molecules are directly induced, in the presence of Marimastat, by VEGF-A that instead plays a passive role in amoeboid angiogenesis despite the activation of its receptor. Since it well known that MMPs can enhance the availability and bioactivity of VEGF and its receptor VEGFR2, the stimulation of angiogenesis through amoeboid cell strategy, regardless of VEGF stimulus, is related to the MMP proteolytic activity inhibition.

## Conclusions

Despite recent improvements, there are still significant challenges to implement anticancer strategies in clinical. Our findings point out the need to consider the crucial role of tumor stroma heterogeneity in cancer progression, in particular the role played by mesenchymal to amoeboid transition in endothelial cells, thereby how their plasticity can contribute to tumor angiogenesis in a unique way that limited the efficacy of MPIs and still limits the effectiveness of Bevacizumab therapy due to their unresponsive to VEGF. Moreover, the in vivo data, besides showing an increase of protease inhibitors with increase of breast cancer progression, also indicate that measuring the amount of protease inhibitors in the serum can be essential to assess the susceptibility of single patients to a possible anti-amoeboid therapy, thus guarantying for the efficacy of the treatment.

Our work clearly challenges the classic dogma stating that the remodeling of extracellular matrix by MMPs is necessary for angiogenesis and metastases and that VEGF can be worthless in some circumstances, as well as during “amoeboid angiogenesis”. We deeply explored this new mechanism underlying the importance to consider it for new anti-cancer drugs.

## Data Availability

All data generated or analyzed during this study are included in this published article. If necessary original data are also available from the corresponding author on reasonable request.
